# The Impact of Delayed Processing of Chilled Whole Blood Specimens on the Measurement of Nutritional Biomarkers in the United Kingdom National Diet and Nutrition Survey Rolling Programme

**DOI:** 10.1016/j.tjnut.2024.07.008

**Published:** 2024-07-14

**Authors:** Kerry S Jones, Sarah R Meadows, Damon A Parkington, Dave Collins, Beverley Bates, Albert Koulman, Polly Page

**Affiliations:** 1 Nutritional Biomarker Laboratory, MRC Epidemiology Unit, University of Cambridge, Cambridge, United Kingdom; 2 Nutrition Measurement Platform, MRC Epidemiology Unit, University of Cambridge, Cambridge, United Kingdom; 3 NatCen Social Research, London, United Kingdom

**Keywords:** micronutrients, biomarkers, preanalytical, vitamin, stability

## Abstract

**Background:**

The logistics of timely processing of blood specimens remains a barrier in population health studies to the generation of micronutrient status data.

**Objectives:**

To test a blood specimen processing protocol that includes overnight postage with cooling and its effect on nutritional biomarker concentrations.

**Methods:**

This study was embedded within the United Kingdom National Diet and Nutrition Survey. Paired specimens were collected from 64 participants (16 y+). One set of specimens were processed within 2 h of collection [“field”] and paired samples were mailed in an insulated box with cold packs using an overnight postal service to a central laboratory [“postal”]. Specimen processing protocols were aligned across field sites and the central laboratory. Specimens were frozen and later analyzed using established methods for vitamins, minerals, lipids, ferritin, and C-reactive protein (CRP). Percent difference was calculated between protocols and compared with quality specifications determined from intra- and interindividual variation.

**Results:**

In the postal protocol, ferritin [geometric mean percent difference (95% confidence interval)] [6% (3, 8)] (*P* = 0.002) and zinc [4% (1, 6)] (*P* = 0.004) were higher compared with the field protocol. Retinol [−3% (−4, −1)] (*P* < 0.0001) and selenium [−3% (−5, −1)] (*P* = 0.003) concentrations were lower in the postal protocol, whereas total [2% (1, 3)] and HDL [4% (2, 5)] cholesterol were higher (*P* < 0.0001) than in the field protocol. Percent differences were within the optimum quality specification for the majority of biomarkers, but ferritin, zinc, and selenium fell outside of the optimum limits. Higher ferritin concentration in the postal protocol led to a decrease in the proportion of specimens with ferritin concentration <15 μg/L from 13% to 9%.

**Conclusions:**

The majority of micronutrient biomarkers, serum lipids, and CRP were minimally affected by delayed processing when cooled. The study suggests acceptable stability of nutritional biomarkers within the described protocol, which can provide accurate data for nutritional biomarkers commonly measured in studies and surveys.

## Introduction

Micronutrient deficiencies are recognized to be a major cause of morbidity and mortality across the globe [1]. Data on micronutrient status from national and subnational surveys are limited. There are growing calls for objective data to better define and determine the prevalence of micronutrient deficiency, to monitor and evaluate public health and micronutrient supplementation and fortification programs, and to investigate relationships between micronutrient status and health outcomes [2]. Several barriers exist to the collection of micronutrient data, especially nationally representative data, including the logistical complexity and cost of collecting, transporting, and processing the blood specimens required for the most accurate and reliable assessment of micronutrient status. In general, established protocols for research studies and surveys recommend that centrifugation and separation of serum or plasma from whole blood should occur within 2 h of blood specimen collection [3]. This can be challenging and cost-prohibitive in national surveys, particularly when covering large geographical areas and/or in countries with sparse laboratory or medical resources for specimen processing. Alternatives to the within 2-h protocol, which maintains specimen integrity and data quality, could help countries to develop and implement viable approaches for cost effective blood sample processing for surveys and studies running across a large geographical space.

Studies that have examined the effect of delayed processing on different micronutrients under specific conditions do not show consistent results. This may be a consequence of the specific conditions tested, the analytical methods used, the biomarkers measured, and the methods of data analysis and presentation [[4], [5], [6], [7], [8]]. Recently, we showed that delayed processing of chilled whole blood specimens for 24 h did not affect the concentration of the majority of micronutrient status markers [3]. However, this study was relatively small and was conducted under highly controlled conditions. To test the feasibility of a protocol that includes a 24-h delay before blood specimen processing, and its effect on nutritional biomarker concentrations, we have conducted a follow-up study embedded within the United Kingdom National Diet and Nutrition Survey (NDNS). In the NDNS Rolling Programme (RP) Years 1 to 11 (2008–2019), blood specimens were collected from participants in their own homes, and blood processing was performed in local field laboratories before freezing [9]. Here, we describe a comparison of the field laboratory processing protocol with the alternative approach that included overnight cooled postage of the blood specimens to a central laboratory for processing.

## Methods

### Setting

The study was conducted between November 2021 and March 2022 and was nested within the United Kingdom NDNS RP, a continuous, cross-sectional survey designed to collect nationally representative data on food consumption, nutrient intakes, and nutritional status. The survey sample is from the general population living in private households and aged 1.5 y and older. Complete details of the survey have been published previously [9]. Ethical permission for the NDNS was obtained from the United Kingdom National Health Service (NHS) Research Authority Research Ethics Committee (REC) and approved by East of England – Cambridgeshire South REC (approval reference 13/EE/0016).

### Substudy

Participants for this substudy were male and female and were restricted to age 16 y and older due to limitations of blood volume collection in younger children. Participants provided informed, written consent. In the NDNS RP, blood specimens are primarily collected from participants after an overnight fast and the same was true for this study with only 2 participants nonfasted. The primary aim of the study was to compare the established NDNS blood processing protocol [10] against a protocol where the specimens were posted overnight to a single, central laboratory [Medical Research Council (MRC) Epidemiology Unit, University of Cambridge].

Venous blood specimens were collected in participants’ homes by biomedical fieldworkers with phlebotomy training. As per the usual NDNS blood sampling protocol, ≤6 blood tubes were collected from each participant, 2 of each of the following: trace element-free serum tubes, lithium heparin (LH) tubes, and EDTA tubes (all tubes were acquired from BD Vacutainers, BD Ltd).

**FIGURE 1:**
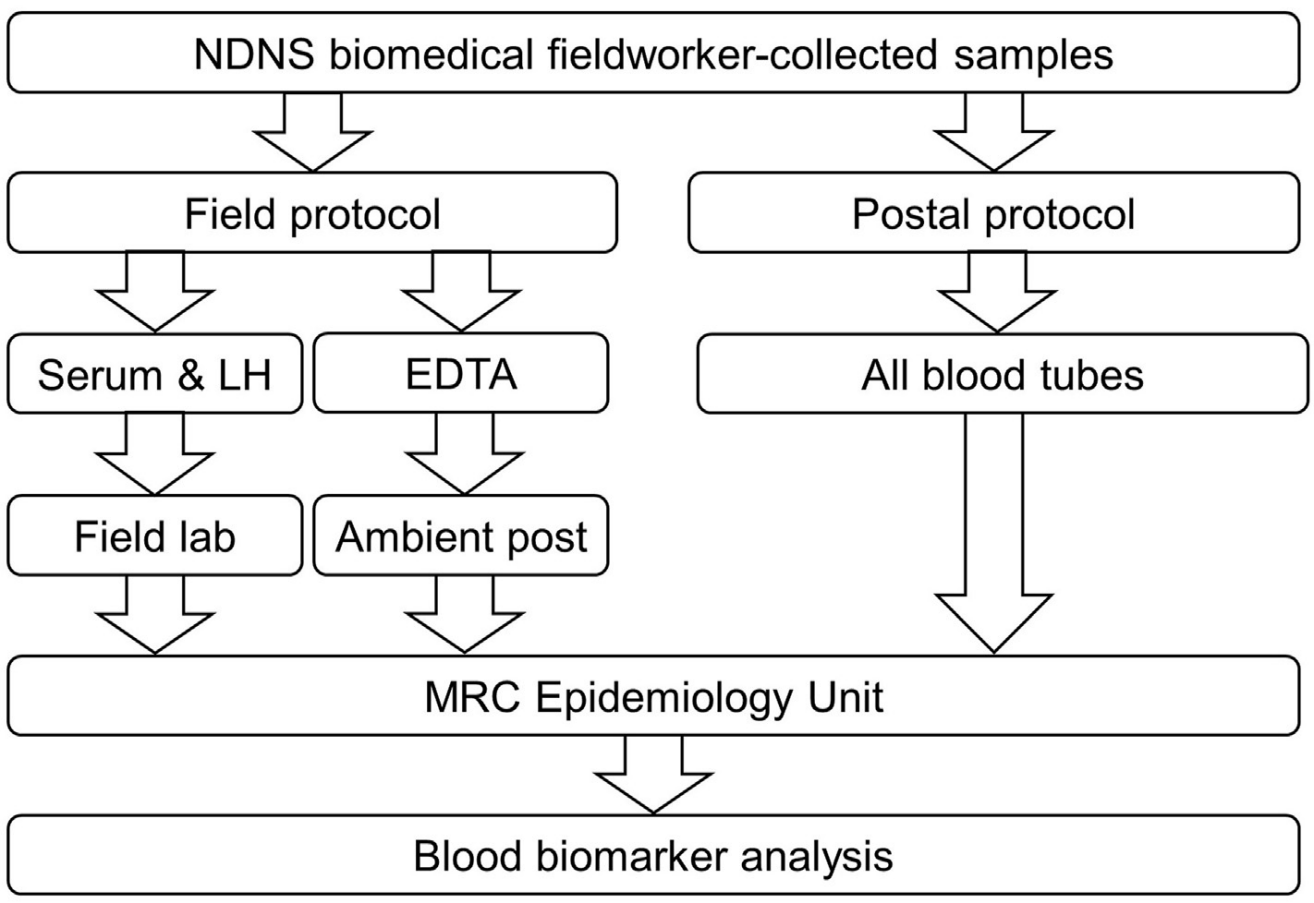
Schematic diagram of field and postal protocols. In the field protocol, EDTA specimens were posted at ambient temperature direct to the MRC Epidemiology Unit; serum and LH tubes were processed in the field laboratory and aliquots returned to the MRC Epidemiology Unit on dry ice before a further thawing and aliquoting before biomarker analysis. In the postal protocol, all specimens were mailed overnight with frozen cold packs and processed on arrival at the MRC Epidemiology Unit the following morning. All specimens were subsequently frozen at −70°C until biomarker analysis. MRC, Medical Research Council.

One set of tubes was processed using established NDNS protocols [9,10] and is subsequently referred to as the “field” protocol. In summary, one serum and one LH tube were packaged in a cold box with cold packs and delivered by the biomedical fieldworker within 2 hours to one of a network of field laboratories recruited as processing laboratories for NDNS – these included hospital and research, as well as private laboratories. For this study, 13 different laboratories were utilized. All field laboratory personnel working on NDNS had training and followed standard operating procedures (SOPs) for NDNS specimen processing. After processing, the samples were stored frozen until dispatch on dry ice to the MRC Epidemiology Unit. The NDNS biomedical fieldworker sent the EDTA tube by ambient post to the MRC Epidemiology Unit (Figure 1).

The other set of tubes (1 serum, 1 LH, and 1 EDTA tube) were sent overnight directly to the MRC Epidemiology Unit; this protocol from hereon is referred to as the “postal” protocol. Specimens were placed in absorbent pouches (#AB010, Alpha Laboratories, UK) and then in clear zip seal bags, wrapped in a double layer of bubble wrap, and placed in a foam insulated box (#GFS-3, Chilled Packaging, UK) containing 2 cold packs frozen overnight in a domestic −20°C freezer. The sealed boxes were taken by the biomedical fieldworker to a local Post Office and sent by Royal Mail guaranteed next-day delivery to the MRC Epidemiology Unit. On arrival the morning after specimen collection, postal specimen temperature was recorded with an infrared thermometer (#830-T4, Testo Ltd, UK), and specimens were processed using the same SOPs to generate a matching set of specimens for analysis. Specimens were frozen at −70°C until analysis.

### Laboratory processing of blood tubes

The same laboratory processing protocols were used for both field and postal specimens. From the EDTA tubes, 100 μL of whole blood was removed and added to 1 mL of 1% w/v ascorbic acid solution. Specimens were frozen at −70°C before shipment on dry ice to the Centers for Disease Control and Prevention (CDC) for whole blood folate analysis. The remaining EDTA whole blood was analyzed for full blood count on the day of receipt at the Pathology Department of Addenbrooke’s Hospital (Cambridge University Hospitals NHS Foundation Trust). Serum and LH whole blood specimens were centrifuged at 2000 × *g* at 4°C for 20 min. Serum and plasma were removed into aliquots in 2 mL microtubes (Sarstedt Ltd) and stored at −70°C. For vitamin C stabilization, 400 μL of LH plasma was added to 400 μL of 10% w/v metaphosphoric acid (MPA).

After the removal of plasma from LH tubes, washed erythrocytes were prepared by adding normal saline so the volume in the tube was approximately equal to the original whole blood volume. The tubes were inverted several times to mix and then subsequently centrifuged at 2000 × *g* at 4°C for 10 min. The supernatant was removed, and the process was repeated 2 more times. Washed erythrocytes were stored at −70°C until analysis for biomarkers of vitamin B1 [erythrocyte transketolase activation coefficient (ETKAC)] and vitamin B2 [erythrocyte glutathione reductase activation coefficient (EGRAC)] [11,12].

Because the “gold standard” for specimen processing is centrifugation within 2 h, the primary aim of this substudy was to compare the effect on nutritional biomarker concentrations of the delay in processing of the postal protocol against the field laboratory protocol. However, because in NDNS the EDTA tube has traditionally been mailed using ambient postage, the results for EDTA-based biomarkers are not included in the main results, but can be found in Supplemental Table 1.

Table 1 [10,[13], [14], [15], [16], [17], [18], [19]] provides a list of analytes and analytical methods. Most analyses were performed at the Nutritional Biomarker Laboratory, MRC Epidemiology Unit, except for whole blood folate (CDC), full blood count and vitamin B12 (Addenbrooke’s Hospital, Cambridge University Hospitals NHS Foundation Trust), serum lipids, ferritin, and C-reactive protein (CRP) (Core Biochemical Assay Laboratory, Cambridge University Hospitals NHS Foundation Trust), and serum zinc and selenium (Trace Element Laboratory, University Hospital Southampton NHS Foundation Trust). Full method details and contemporaneous quality control and quality assurance data are available elsewhere [3,10]. In addition, to standardized measurement of hemolysis, the hemolysis index was assessed in serum specimens using a Siemens Dimension EXL and based on absorbance at wavelengths to detect the presence of hemoglobin in the specimen.

**TABLE 1:** Assay analytical variation and inter- and intraindividual variation used to calculate quality specifications

Analyte	Matrix	Method	Variation (%)			Quality specifications (%)^4^		
			Analytical^1^	Interindividual^2^	Intraindividual^3^ [reference]	Optimum	Desirable	Minimum
Clinical markers								
C-reactive protein	Serum	Siemens Dimension Xpand Analyser^5^	1.7	76.3	42.2 [13]	10.4	20.8	31.3
Ferritin	Serum		4.2	15.0	14.9 [14]	2.7	5.3	8.0
Triglycerides	Serum		0.9	56.8	28.8 [14]	7.5	14.9	22.4
HDL cholesterol	Serum		1.6	28.3	12.4 [14]	4.2	8.3	12.5
Total cholesterol	Serum		1.2	22.3	8.2 [14]	2.9	5.9	8.8
Fat-soluble vitamins								
25-Hydroxyvitamin D	Serum	LC-MS/MS	3.3	37.9	11.3 [13]	6.1	12.1	18.2
Retinol	Plasma	HPLC with PDA detection	4.1	29.2	9.5 [14]	3.5	6.9	10.4
α-Tocopherol	Plasma		3.6	35.1	11.3 [14]	3.5	6.9	10.4
γ-Tocopherol	Plasma		6.7	51.0	12.1 [14]	5.6	11.1	16.7
Lutein	Plasma		5.7	46.0	17.4 [14]	6.8	13.6	20.5
Lycopene	Plasma		7.5	47.4	26.1 [14]	7.2	14.3	21.5
β-Carotene	Plasma		5.6	67.4	24.2 [14]	9.4	18.7	28.1
Minerals								
Selenium	Serum	ICP-MS	5.6	13.2	5.1 [14]	2.2	4.4	6.6
Zinc	Serum		4.2	12.7	9.3 [15]	2.1	4.3	6.4
Water-soluble vitamins								
ETKAC (vitamin B1)	wRBC	Enzymatic method	2.5	4.8	3.7 [16]	0.8	1.6	2.4
EGRAC (vitamin B2)	wRBC		1.5	13.7	3.2 [16]	1.8	3.5	5.3
PLP (vitamin B6)	Plasma	HPLC with fluorometric detection	5.0	34.0	20 [13]	7.6	15.3	22.9
PA (vitamin B6)	Plasma		5.0	66.8	67.0 [17]	10.3	20.7	31.0
Folate	Serum	LC-MS/MS	2.8	64.3	22.6 [15]	7.8	15.7	23.5
Vitamin B12	Serum	Advia Centaur (immunoassay)^5^	3.6	43.6	13.4 [14]	4.6	9.3	13.9
Holotranscobalamin	Serum	ELISA^6^	4.2	41.8	13.0 [18]	55.4	10.7	16.1
Vitamin C	Plasma	Fluorescence method	3.4	31.0	26.0 [14]	6.8	13.5	20.3

Abbreviations: EGRAC, erythrocyte glutathione reductase activation coefficient; ETKAC, erythrocyte transketolase activation coefficient; HPLC, high-performance liquid chromatography; PA, 4-pyridoxic acid; PLP, pyridoxal-5-phosphate; PDA, photo diode array detector; ICP-MS, inductively coupled plasma mass spectrometry; LC-MS/MS, liquid chromatography-tandem mass spectrometry; wRBC, washed red blood cells (collected in lithium heparin tube).

1 Analytical precision was obtained for methods as used for United Kingdom National Diet and Nutrition Survey Rolling Programme [10].

2 Interindividual variation was obtained from National Diet and Nutrition Survey Rolling Programme [10].

3 Intraindividual variation was obtained from sources described in the table.

4 Quality specifications were derived from inter- and intraindividual variation [19].

5 Siemens Healthcare Limited, Surrey, United Kingdom.

6 Abbott, Maidenhead, United Kingdom.

### Sample size

The sample size was calculated based on the ability to detect a 4% difference with 80% power at the 5% significance level and was reliant on estimates of within-person variability, which were obtained from a variety of sources (Table 1 [10,[13], [14], [15], [16], [17], [18], [19]]). A minimum of 53 pairs of specimens (or fewer pairs for some analytes) were required to detect a 4% difference in a selection of key analytes, including vitamin D, folate, vitamin B6, vitamin E, and vitamin C. To detect the same difference for retinol or β-carotene would have required 158 and 233 specimen pairs, respectively, thus the choice of 53 pairs of specimens was a compromise considering cost and feasibility and the uncertainty around the estimates of within-person variability on which the sample size was based.

### Statistical analysis

To normalize skewed data and to allow calculation of the percent difference between postal and field laboratory protocol specimens, analysis was performed using log-transformed values. Only paired results were included for each analyte and several data points were excluded from data analysis, either due to extreme values, concentrations less than the limit of quantitation, or specimen hemolysis. Nonfasted participants were included in data analysis. Details of exclusions are provided in Supplemental Table 2. The percent difference between the geometric mean (95% confidence interval [CI]) concentrations was calculated by *1)* exponentiation of the numerical difference between the groups to obtain the ratio difference and *2)* calculation of percent difference: (ratio difference – 1) × 100. Differences between processing protocols were tested with paired *t*-test, and the result was considered significant where *P* < 0.05.

Percent differences in analyte concentrations between postal and field laboratory specimens were compared against change limits (optimum, desirable, or minimum performance) calculated from intra- and interindividual variation [13,19] and described in Table 1 [10,[13], [14], [15], [16], [17], [18], [19]]. Interindividual variation was calculated from NDNS year 9 to 11 (2016–2019) data from adults aged 16 y and over. Outliers were eliminated based on the Tukey method [14]. Analytical precision is described for methods obtained during analysis in NDNS [10].

## Results

**FIGURE 2:**
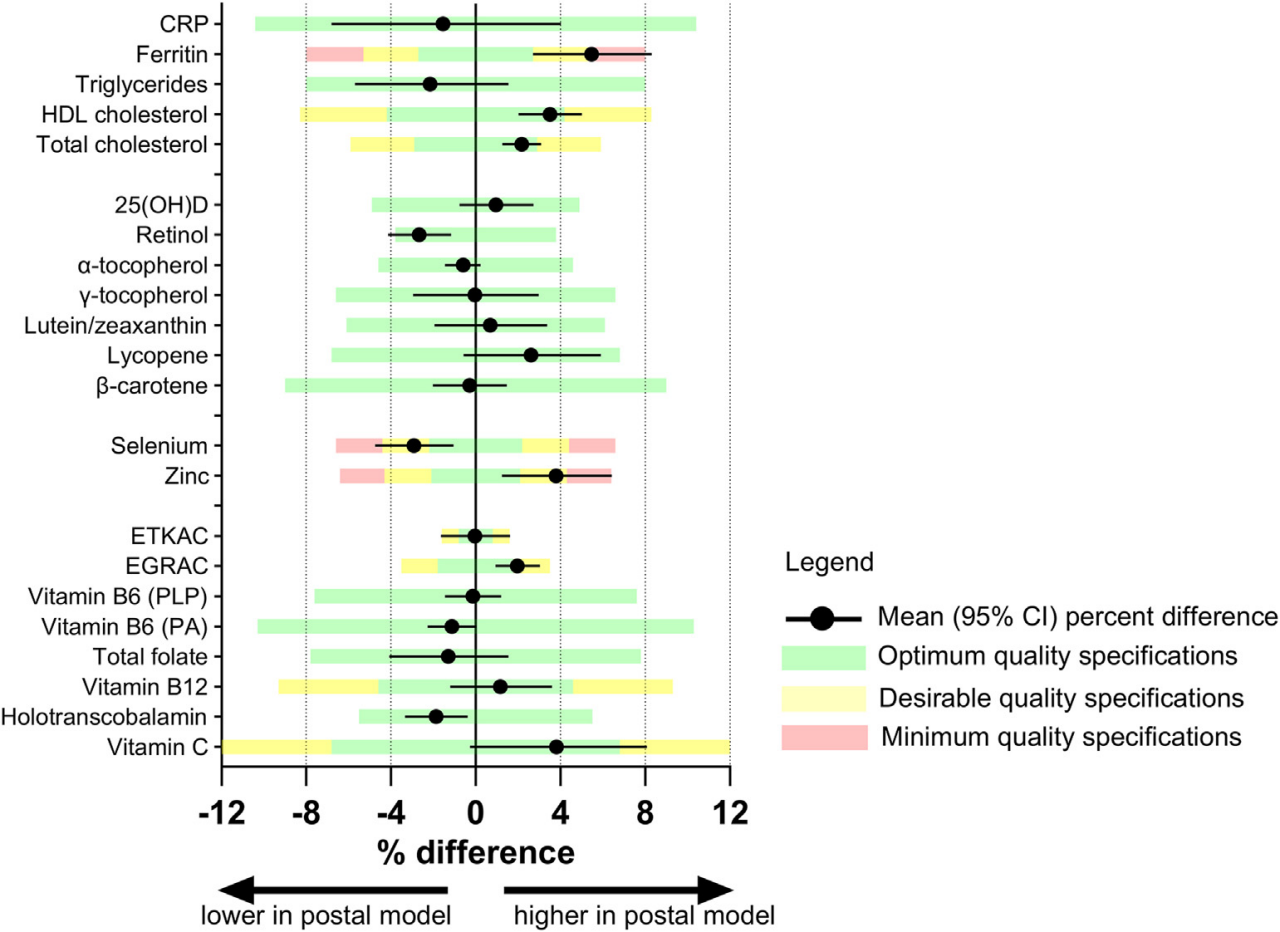
Geometric mean percent difference (95% confidence interval) between the postal and field laboratory protocols in relation to quality specifications calculated from inter- and intraindividual variation.

**FIGURE 3:**
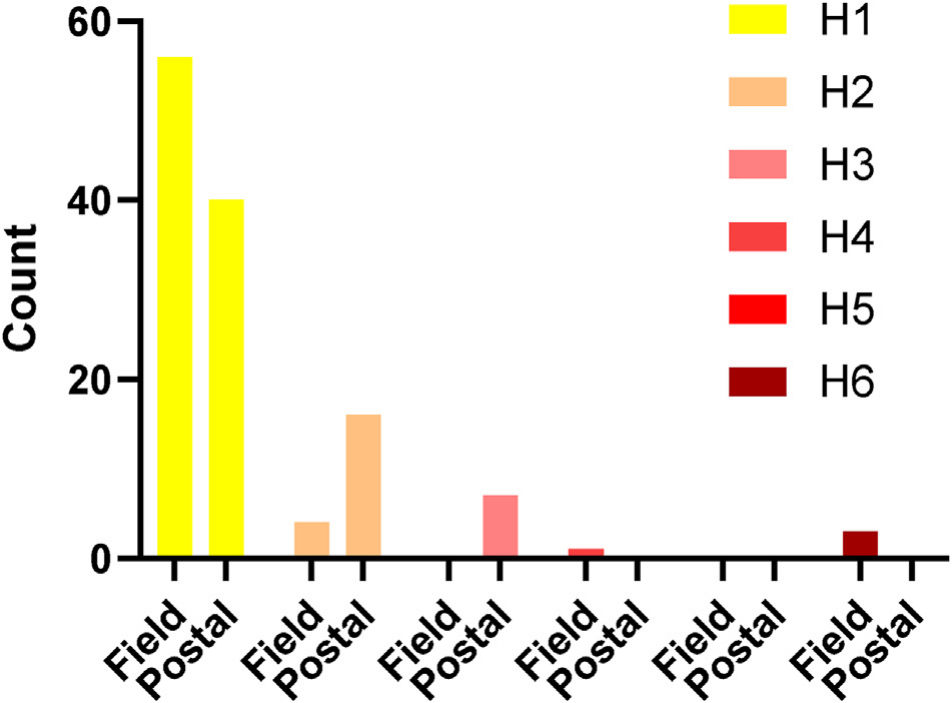
Hemolysis scores of field and postal specimens. H-score indicates the degree of hemolysis (H1 lowest to H6 highest) measured on serum specimens using Siemens Dimension EXL Analyzer.

Concentration data and differences between field and postal protocols are presented in Table 2. Figure 2 provides a graphical representation of the percent difference in the context of the change limits. Differences between processing protocols were mostly small or modest. Percent differences between biomarker concentrations in each protocol were generally <4% and within quality specifications derived from intra- and interindividual variation. Ferritin (6%) and zinc (4%) were higher in the postal protocol compared with the field laboratory protocol, and selenium was lower (3%) in the postal protocol. In the postal protocol, total cholesterol (2%) and HDL cholesterol (4%) were marginally higher, and triglycerides were marginally lower than in the field laboratory protocol. Retinol was 3% lower with the postal protocol. Table 3 indicates the proportion of specimens with values above or below commonly used thresholds to indicate risk of deficiency or sufficiency. The effect of the postal protocol on the proportions relative to deficiency cutoffs was also small. For most analytes, the proportions in the postal protocol specimens were within 2%–3% observed in the field laboratory protocol specimens. Higher ferritin in the postal protocol led to a decrease in the proportion of specimens with ferritin concentration <15 μg/L from 13% to 9%. The time (h) from specimen collection to unpacking of the postal specimens at the MRC Epidemiology Unit was mean (SD) 24.1 (1.4) h, and the temperature (°C) of the postal specimens when unpackaged was 9 (2)°C. The hemolysis index indicated a slightly higher degree of mild hemolysis (25 to ≤50 mg/dL) in the postal protocol specimens (Figure 3).

**TABLE 2:** Analyte concentrations and percent difference between field laboratory and postal protocols

		Geometric mean (95% confidence interval)			
	*n* pairs	Field	Postal	Percent difference from field	*P*
Clinical markers					
C-reactive protein, mg/L	64	1.23 (0.94, 1.62)	1.21 (0.92, 1.60)	−1.5 (−6.8, 4)	0.6
Ferritin, μg/L	64	66.7 (51.1, 86.9)	70.3 (54.5, 90.7)	5.5 (2.7, 8.3)	0.002
Triglycerides, mmol/L	61	1.02 (0.91, 1.15)	1.00 (0.89, 1.13)	−2.1 (−5.7, 1.5)	0.2
HDL cholesterol, mmol/L	64	1.40 (1.31, 1.50)	1.45 (1.36, 1.55)	3.5 (2.02, 5.01)	<0.0001
Total cholesterol, mmol/L	61	4.77 (4.50, 5.06)	4.87 (4.59, 5.18)	2.2 (1.3, 3.1)	<0.0001
Fat-soluble vitamins					
25-Hydroxyvitamin D, nmol/L	64	42.6 (37.4, 48.5)	43.0 (37.9, 48.8)	0.96 (−0.8, 2.7)	0.3
Retinol, μmol/L	57	1.98 (1.84, 2.14)	1.93 (1.79, 2.08)	−2.7 (−4.1, −1.2)	0.0009
α-Tocopherol, μmol/L	57	29.8 (27.8, 31.9)	29.6 (27.6, 31.8)	−0.6 (−1.4, 0.2)	0.2
γ-Tocopherol, μmol/L	57	1.42 (1.25, 1.62)	1.42 (1.24, 1.62)	−0.03 (−3, 3)	0.98
Lutein, μmol/L	57	0.22 (0.20, 0.25)	0.22 (0.20, 0.25)	0.7 (−1.9, 3.4)	0.6
Lycopene, μmol/L	57	0.82(0.70, 0.95)	0.84 (0.73, 0.97)	2.6 (−0.6, 5.9)	0.1
β-Carotene, μmol/L	56	0.51 (0.42, 0.61)	0.50 (0.42, 0.60)	−0.3 (−2, 1.5)	0.7
Minerals					
Selenium, μg/L	57	1.08 (1.03, 1.13)	1.05 (0.99, 1.10)	−2.9 (−4.7, −1.05)	0.003
Zinc, μg/L	51	13.7 (13.3, 14.2)	14.3 (13.7, 14.9)	3.8 (1.2, 6.43)	0.004
Water-soluble vitamins					
ETKAC (B1) [ratio]	62	1.11 (1.10, 1.12)	1.11 (1.10, 1.12)	−0.025 (−1.6, 1.62)	0.98
EGRAC (B2) [ratio]	63	1.39 (1.34, 1.44)	1.42 (1.37, 1.47)	1.97 (0.93, 3.03)	0.0003
Vitamin B6 (PLP), nmol/L	57	35.0 (29.4, 41.7)	35.0 (29.5, 41.5)	−0.1 (−1.5, 1.2)	0.8
Vitamin B6 (PA), nmol/L	56	22.8 (20.2, 25.7)	22.6 (20.0, 25.5)	−1.1 (−2.3, 0.03)	0.06
Total folate, nmol/L	63	14.2 (11.7, 17.1)	14.0 (11.6, 16.8)	−1.3 (−4.1, 1.5)	0.4
Vitamin B12, ng/L	57	391 (348, 440)	396 (351, 446)	1.2 (−1.2, 3.61)	0.3
Holotranscobalamin, pmol/L	57	72.8 (63.4, 83.5)	71.4 (62.4, 81.8)	−1.9 (−3.3, −0.4)	0.02
Vitamin C, μmol/L	42	41.9 (34.5, 50.7)	43.5 (36.3, 52.0)	3.8 (−0.27, 8.1)	0.07

Abbreviations: EGRAC, erythrocyte glutathione reductase activation coefficient; ETKAC, erythrocyte transketolase activation coefficient; PA, 4-pyridoxic acid; PLP, pyridoxal-5-phosphate.

**TABLE 3:** Proportion of results against accepted cutoffs

		% Beyond cutoff	
Analyte	Cutoff	Field protocol	Postal protocol
Ferritin	<15 μg/L	13	9
Triglycerides	<1.7 mmol/L	80	80
HDL cholesterol	>1 mmol/L	88	89
Cholesterol	<5 mmol/L	51	48
25(OH)D	<25 nmol/L	14	13
Retinol	<1.05 μmol/L	2	2
Serum zinc	<11 μmol/L	0	2
ETKAC	>1.25	0	0
EGRAC	>1.3 (ratio)	62	67
Serum total folate	<7 nmol/L	13	15
Vitamin B12	<150 ng/mL	2	2
Vitamin C	<11 μmol/L	2	2

Abbreviations: EGRAC, erythrocyte glutathione reductase activation coefficient; ETKAC, erythrocyte transketolase activation coefficient.

## Discussion

This study compared two different protocols for blood specimen processing as applied in the United Kingdom NDNS. Blood specimens were either processed in field laboratories, located within 2 h of where the blood specimen was collected, or mailed, cooled, to a central laboratory for processing using an overnight postal delivery service. Laboratory processing included centrifugation and aliquoting, and for specific assays, the addition of MPA for future analysis of vitamin C, and washing of red blood cells for ETKAC and EGRAC analysis.

Overall, the differences between processing protocols were small, and the impact of delayed sample processing associated with the postal protocol was typically within 3% of the reference, i.e., the tube processed within 2 h, and within quality specifications. This was true for vitamin D, retinol, vitamin E, vitamin B6, vitamin B12, and serum folate. The effect of the postal protocol on the proportions relative to deficiency cutoffs was also small and for the majority of analytes within 1%–2% of the value in field laboratory processed specimens. The largest difference was for ferritin where this resulted in a 4% lower proportion of participants with ferritin <15 μg/L. This is in slight contrast to our earlier study where we observed a <1% concentration difference for ferritin with delayed sample processing [3]. A systematic review concluded that ferritin was stable in whole blood at room temperature for ≤24 h [20], but other studies have observed increased ferritin concentrations under such conditions. For example, Drammeh et al. [5] observed a 9% increase in ferritin concentration after 1-d storage of whole blood at 32°C, van Eijsden et al. [6], a 2%–3% increase after 26–28 h at room temperature, and Abraham et al. [21], a >10% increase after 24 h at room temperature [21]. Collectively, data from these studies, including the current study, and other reports [22,23], suggests that the increase in ferritin concentration associated with delayed processing can be ameliorated by cooling specimens.

An increase in serum zinc concentration with delayed processing of the postal protocol is consistent with other studies; zinc concentration reportedly increased by ≤20% when whole blood was kept at room temperature for 24 h [21,24,25], whereas selenium concentrations were reported as stable [24]. As for ferritin, cooling of whole blood appears to mitigate the impact of delayed processing as the increase in zinc concentration was limited in this, and our previous study to 4% or less [3]. This is supported by other studies that have observed similar changes to ours and a lower increase in zinc concentration with cooling [26,27]. The increase in zinc concentration with delayed processing appears to be related to contact time between serum and red blood cells because after centrifugation and storage of serum or plasma, zinc concentrations remain relatively stable [26,28]. Higher zinc concentration may also be related to the marginally higher degree of mild hemolysis (hemolysis score ≤ 3) as measured in the postal specimens.

We compared the percent difference between sample processing protocols against change limits calculated from intra- and interindividual variation, and this is one way to determine the biological, rather than statistical significance of an observed change. Normal biological variation, due to, for example, fasting status, particularly for micronutrients may be considerably greater than the magnitude of the sample processing-related effects we observed here. National surveys, due to reasons of inclusion, pragmatism, and burden, typically collect blood specimens from a mixture of fasted and nonfasted participants. However, systematic effects in one direction may have a material impact on the concentration data and consequently data interpretation. Understanding and considering the relative size of these different effects therefore becomes relevant. Comparisons against the analytical variation are an alternative method, and analytical %CVs (described in Table 1), for ferritin and zinc are both 4.2%. In addition, the 95% CI of the percent difference also needs to be considered. A percent difference with a tight 95% CI, together with a small analytical %CV suggests a consistent and repeatable effect (e.g., as observed for total cholesterol in this study). In contrast, a wider confidence interval would suggest more variability in the effect of protocol differences (e.g., as observed for CRP).

Compared with our previous study, which was performed under controlled laboratory conditions, this study was performed nested within ongoing NDNS fieldwork that provided a field experiment that took into account the myriad of variables that can influence specimen processing quality. Both nonfasted and fasted participants were included in the data analysis; the analysis was based on paired samples and biomarker concentrations for nonfasted participants were within the range of fasted participants. It is well documented that preanalytical factors can have a major influence on results and that they should be considered, together with analytical factors, in the full cycle of sample collection and processing. In this study, 13 different field laboratories were involved in specimen processing. Although all laboratory staff were provided training and followed SOPs, variation between laboratories and personnel in specimen processing is inevitable in multicenter studies. In contrast, the postal protocol specimen processing was centralized in a single laboratory, likely providing advantages for the standardization of procedures because constant monitoring is possible and more frequent and regular processing of specimens reinforces procedures. Any issues in specimen quality can be detected and acted upon more rapidly.

The strengths of this study include the sample size for this study type, the use of standardized protocols for specimen processing and measurement with well-described, high-quality analytical methods, and the realistic rather than laboratory-controlled setting that includes the complexities and challenges characteristic of field work using different laboratories. However, the specimen processing protocols are specific to the United Kingdom NDNS, and conclusions from this study may not hold true for all settings. In addition, results may be different in other populations with different levels of micronutrient deficiencies. Nevertheless, the study provides useful data on the relative stability of nutritional biomarkers applicable to both surveys and multicenter studies.

Nutritional surveys are complex, logistically challenging, and expensive, and tradeoffs between logistics, feasibility, cost, and quality can be necessary. As recently discussed in the context of NHANES, solutions to improve the cost-effectiveness of surveys are necessary as government budgets come under increasing pressure [29]. The collection and measurement of biological specimens is by its nature expensive. However, nutritional biomarker analysis remains an essential component of national surveys and provides government, national, and international agencies and other stakeholders with necessary data to understand the extent of, and change in nutritional status, or to plan and monitor the impact of public health interventions [2], for example, in the context of the folic acid fortification of flour [30,31]. It is therefore vital that evidence is available to guide pragmatic, affordable, and effective protocol development. The results from this study may be relevant to any survey or multicenter study that has to consider the logistics of collecting and transporting specimens from multiple sites. Other specimen types such as dried blood spots (DBS) or microsampling devices offer alternative sample collection strategies where storage of venous blood specimens is problematic. DBS can offer many advantages over venous blood samples including simpler collection protocols, smaller sample volume, and easier transportation, often not requiring a cold chain. However, notwithstanding potential differences between analyte concentrations in venous and capillary specimens (e.g., for ferritin [32,33], zinc [27], and retinol [34]), factors such as DBS quality, time, and temperature can affect analyte stability [35,36], including for micronutrients [34].

In conclusion, few studies have examined the effect of delayed processing of refrigerated or chilled whole blood, and/or have focused on specimens stored at room temperature for varying time periods. In this study, most biomarkers were minimally affected by the 24-h processing delay when maintained at ≤10°C. The 24-h processing delay associated with the chilled postal protocol in this study had the greatest impact on ferritin and zinc. This observation is consistent with previous studies on these biomarkers, but the current study suggests that instability for these analytes can be mitigated when the specimens are shipped with cold packs and temperature maintained at ≤10°C. Overall, the results show that the 24-h processing delay with specimens maintained at ≤10°C (within the above protocol) can deliver accurate data and is applicable in surveys and studies.

## Acknowledgments

We would like to thank Amanda McKillion and Tabasum Tabasum for sample analysis at Nutritional Biomarker Laboratory, MRC Epidemiology Unit, University of Cambridge, United Kingdom; Steve Knighton, Samuel Odeyemi, Carol Dorling, Lydia Moore, Dani Ghosh at MRC Epidemiology Unit, University of Cambridge, United Kingdom, for receipt and processing of specimens; and Steph Moore and Jackie Foreman at MRC Epidemiology Unit, University of Cambridge, United Kingdom, for study support. We also thank NatCen biomedical fieldworkers who collected and dispatched the samples, and field laboratory staff for sample processing. Finally, we express our appreciation to all NDNS participants.

## Author contributions

The authors’ responsibilities were as follows – KSJ, SRM, DAP, DC, BB, AK, PP: designed and conducted the research; KSJ, DC: analyzed the data; KSJ: wrote the article; KSJ: responsible for the final content; and all authors: read and approved the final manuscript.

## Conflict of interest

The authors report no conflicts of interest.

## Funding

The United Kingdom National Diet and Nutrition Survey has jointly funded by the Office for Health Improvement and Disparities (Department of Health and Social Care Office for Health) and the Food Standards Agency and is conducted by NatCen Social Research working with the MRC Epidemiology Unit at the University of Cambridge, United Kingdom. This research was also supported by the NIHR Cambridge Biomedical Research Center (NIHR203312). The views expressed are those of the authors and not necessarily those of the NIHR or the Department of Health and Social Care. The NIHR Cambridge Biomedical Research Center is a partnership between Cambridge University Hospitals NHS Foundation Trust and the University of Cambridge, funded by the NIHR. The views expressed are those of the authors and not necessarily those of the NHS, the NIHR, or the Department of Health and Social Care.

For the purpose of Open Access, the author has applied a Creative Commons Attribution (CC BY) license to any author accepted manuscript version arising.

## Data availability

Data described in the manuscript can be made available upon request.
