# Corrigendum to ‘The Impact of Delayed Processing of Chilled Whole Blood Specimens on the Measurement of Nutritional Biomarkers in the United Kingdom National Diet and Nutrition Survey Rolling Programme’,[J Nutr 154 (2024) 2818–2826]

**DOI:** 10.1016/j.tjnut.2025.03.030

**Published:** 2025-04-11

**Authors:** KS Jones, SR Meadows, DA Parkington, D Collins, B Bates, A Koulman, P Page

**Affiliations:** 1 Nutritional Biomarker Laboratory, MRC Epidemiology Unit, University of Cambridge, Cambridge, United Kingdom; 2 Nutrition Measurement Platform, MRC Epidemiology Unit, University of Cambridge, Cambridge, United Kingdom; 3 NatCen Social Research, London, United Kingdom

The authors regret that in Table 2 there was an error in the concentration units for selenium and zinc. The correct units for selenium and zinc concentration are μmol/L.

The authors apologize for any inconvenience caused.

