# Supplementary material for: The Impact of Delayed Processing of Chilled Whole Blood Specimens on the Measurement of Nutritional Biomarkers in the United Kingdom National Diet and Nutrition Survey Rolling Programme
Source: J Nutr. 2024 Jul 14;154(9):2818–26. doi: 10.1016/j.tjnut.2024.07.008 (PMC11393162; doi:10.1016/j.tjnut.2024.07.008)
Supplement: multimedia component [file mmc1.docx]

**The impact of delayed processing of chilled whole blood specimens on nutritional biomarkers in the UK National Diet and Nutrition Survey**

Kerry S. Jones, Sarah R. Meadows, Damon A. Parkington, Dave Collins, Beverley Bates, Albert Koulman and Polly Page

**SUPPLEMENTARY MATERIAL**

**Supplementary Table 1.** Red cell folate and hematological indictors for the field lab sample (mailed at ambient conditions) and postal (chilled, overnight) protocol concentrations and percent difference between field lab and postal protocols. The results indicate that cooling of the sample is associated with improved stability for red cell folate and is partly attributable to improved stability of the hematocrit measurement.

|  |  | Geometric mean (95% confidence interval) | | |  |
| --- | --- | --- | --- | --- | --- |
|  | n pairs | Field lab | Postal | Percent difference from field lab | P |
| Red cell folate, nmol/L | 58 | 580 (513, 656) | 631 (556, 715) | 8.7 (5.5, 11.96) | <0.0001 |
| Red cell folate < 2 days post only | 38 | 620 (531, 725) | 654 (558, 765) | 5.4 (2.5, 8.35) | 0.0005 |
| Hematocrit, L/L | 58 | 0.44 (0.43, 0.45) | 0.42 (0.41, 0.43) | -4.7 (-5.9, -3.5) | <0.0001 |
| Hemoglobin, g/dL | 57 | 140 (137, 143) | 141 (138, 144) | 0.7 (0, 1.4) | <0.04 |
| Mean cell volume, fL | 59 | 94 (92, 96) | 89 (88, 91) | -4.8 (-5.5, -4.1) | <0.0001 |
| WBC count x 10^9/L | 58 | 5.72 (5.35, 6.11) | 5.36 (5.01, 5.73) | -6.3 (-9.6, -2.9) | 0.0006 |

**Supplementary Table 2. List of specimen exclusions**

| **Analyte** | **n pairs** | **Exclusions and reason** | **Notes** |
| --- | --- | --- | --- |
| CRP | 64 | None |  |
| Ferritin | 64 | None |  |
| Triglycerides | 61 | n3 specimens for haemolysis score of 6 | Recommendation from manufacturer |
| HDL cholesterol | 64 | None |  |
| Cholesterol | 61 | n3 specimens for haemolysis score of 6 | Recommendation from manufacturer |
| 25(OH)D | 64 | None |  |
| Retinol | 57 | None |  |
| α-tocopherol | 57 | None |  |
| γ-tocopherol | 57 | None |  |
| Lutein/zeaxanthin | 57 | None |  |
| Lycopene | 57 | None |  |
| β-carotene | 56 | 1 pair < assay LOQ |  |
|  |  |  |  |
|  |  |  |  |
| Serum selenium | 57 | None |  |
| Serum zinc | 51 | n3  n2 outlier pairs | Gross haemolysis, not analysed, all field lab specimens  Without exclusion mean difference is 1.7% (reported difference, 3.8%) |
| ETKAC | 62 | None |  |
| EGRAC | 63 | None |  |
| Vitamin B6 (PLP) | 57 | None |  |
| Vitamin B6 (PA) | 56 | 1 pair high | Excluded values: field 163, postal 162 nmol/L |
| Serum total folate | 63 | 1 pair high | Excluded values: field 781, postal 805 nmol/L |
| Vitamin B12 | 57 | n3 specimens for haemolysis score of 6 | Recommendation from manufacturer |
| Holotranscobalamin | 57 | None |  |
| Vitamin C | 42 | n2 below assay LOQ; n6 no MPA in tube; n12 tube weight discrepancy; n5 field lab issue | Including all specimens (n57) except specimens <LOQ and no MPA, mean difference increased to 11.7% from 4.4% |
